# Effectiveness of a mobile health intervention on infant and young child feeding among children ≤ 24 months of age in rural Islamabad over six months duration

**DOI:** 10.12688/f1000research.17037.3

**Published:** 2019-10-14

**Authors:** Subhana Akber, Hana Mahmood, Razia Fatima, Ahmed Wali, Ashraful Alam, Syed Yahya Sheraz, Aashifa Yaqoob, Hina Najmi, Saleem Abbasi, Humaira Mahmood, Michael J. Dibley, Tabish Hazir

**Affiliations:** 1 Maternal, Neonatal and Child Health Research Network, Islamabad, Pakistan; 2 National TB Control Programme, Islamabad, Pakistan; 3 Provincial TB Control Program Balochistan, Quetta, Pakistan; 4 School of Public Health, University of Sydney, Sydney, Australia; 5 International Research Force, Islamabad, Pakistan; 6 MNCH, Sukh Initiative, Karachi, Pakistan

**Keywords:** Mobile health, mhealth, IYCF nutrition, Operational research, Islamabad, Pakistan

## Abstract

**Background: **Childhood development is highly influenced by feeding practices at infancy and young age of the children. Unfortunately, according to the National Nutrition Survey (2011), the prevalence of exclusive breastfeeding in Pakistan was 21% at four months, and 13% at six months of age with 51.3% of mothers initiating semisolid foods to their children at the recommended 6-8 months of age. Pakistan Demographic & Health Survey (PDHS 2018) however; indicates that only 48% of infants are exclusively breastfed which has been improved from 38% as reported in the past five years but still more improvement is envisaged.

**Methods: **A quasi-experimental study design was employed for this post-intervention survey assessing effectiveness of mobile health (mhealth) regarding infant & young child feeding (IYCF) among pregnant and lactating mothers in Tarlai, Islamabad from May to June 2018. A total of 135 mothers who were earlier included in the intervention phase were recruited after obtaining verbal & written consent. The data was entered in EpiData (3.1) and analyzed in SPSS version 21.

**Results: **The mean age of these pregnant and lactating mothers was 30.5 years ± 4.5 SD with the majority of mothers in the age group of 25 to 29 years. After intervention, the overall knowledge of mothers regarding IYCF nutrition was raised among 94 mothers (69.6%) as compared to 74 (54.8%) mothers prior to the intervention. Overall attitude regarding IYCF was found to be positive among 86 (63.7%) of the mothers, whereas 88 (65.2%) of the mothers had good IYCF related practices.

**Conclusion: **Our post-intervention survey signifies the effectiveness of mhealth in raising knowledge, attitude, and practices of mothers regarding IYCF in rural Islamabad. However, implementation of mhealth in masses requires future research specifically to address cost-effectiveness of such interventions in maternal & child health programmes.

## Introduction

Childhood under-nutrition is a major public health problem which has been contributing extensively to childhood mortality and morbidity ^1^. Globally; 45% of child mortality results from *‘undernutrition’* which highlights the right of every child to good nutrition. According to World Health Organization (2018), globally more than 100 million children were found to be stunted, and nearly 52 million were found to be wasted in 2016 alone. Adequate nutrition is required for optimal growth and development of children ^2^. Evidence indicates that under-nutrition leads to severe cognitive and behavioural disabilities throughout life if not managed in early infancy ^3– 5^. The magnitude of malnutrition is extensive in the South Asian region leading to high rates of stunting, wasting, and disease burden ^5^. One of the major causes of these high indicators of undernutrition is poor infant and young child feeding (IYCF) practices. The WHO and United Nations International Children’s Emergency Fund (UNICEF) recommends early initiation of breastfeeding within an hour after birth, and exclusive breastfeeding for the first 6 months during infancy with timely and appropriate initiation of complementary feeding ^6^. Despite this recommendation, the recognized adverse effects of malnutrition and undernutrition among infants and children have been significantly reported in various studies ^7– 9^.

Regarding infant and young child feeding practices, around 40% of infants from 0–6 months of age had been exclusively breastfed, worldwide ^6^. Whereas, only a few children acquire adequate nutrition along with proper complementary feeding which is appropriate for their age group they belong to ^6^. In low-and middle-income countries (LMICs), these sub-optimal feeding practices of infants and young children contribute to the prevailing burden of malnourishment. According to the Pakistan Demographic Health Survey (PDHS, 2018), 38% of children under five years of age are stunted and 17% are severely stunted. However; the national findings also indicate an improved nutritional status of children which has resulted in the decline of stunting from 45% to 38% among children as reported by previous findings of PDHS 2012–2013 ^2, 10, 11^. Lack of knowledge, lower socio-economic status, and relatively low levels of education of mothers or caregivers can be attributed to suboptimal IYCF practices ^12^.

Emphasis has been laid on implementing effective innovative interventions to improve nutrition among children particularly in poor resource countries. One of the most effective strategies laid down by WHO to improve IYCF practices is effective counseling on proper nutritional practices through community health workers ^13, 14^. In Pakistan, these community health workers are referred to as Lady Health Workers (LHWs), recruited under the National Program for Family Planning and Primary Health Care ^15^. With support from WHO, the government of Pakistan launched the ‘Lady Health Workers Programme’ in 1994, which was mainly aimed to provide an effective grassroot level system for accessing primary health care ^15^. This program was aspired to bridge the communities for accessing primary healthcare through LHWs. Moreover; among the various roles and responsibilities under this program, the LHWs are also expected to provide nutritional counseling. However, the deliverables by LHWs somehow are affected due to being overburdened ^16^.

Thus, it is imperative that a facilitating system if is provided to these LHWs will help to reduce their workload. Among some of the innovative strategies of providing health service, mobile health or mhealth, is gaining momentum in low- and middle-income countries. As defined by WHO mhealth is the “provision of health services and information via mobile and wireless technologies” ^17^. The innovation and use of information and technology through mhealth has been vastly employed to address access, resource utilization, and coverage gaps. Many LMICs including those in South Asia have been employing mhealth approach through the Community Health Workers or peer counselors to improve healthcare as an innovative strategy ^18, 19^. A study conducted in Bangladesh demonstrated gaps in IYCF related service delivery which prompted the need of healthcare messages, including information related to emergency and medical care, to be delivered through mobile phones. The potential benefits and necessity of mhealth led the technology to embrace community-based nutrition services to improve the service delivery and coverage related to IYCF nutrition. Mass-scale behavioural interventions that actively included social mobilization at the community-level, media campaigns, and counseling by trained workers have also been found useful ^19– 21^. Evidence from India suggests that IYCF related nutrition among children can be improved using counseling strategies aimed at the parents ^22^. Limited evidence is available from Pakistan indicating effectiveness of mhealth related to IYCF nutrition as mhealth has not been extensively opted for IYCF nutrition solely. Although there lies significant opportunities including use of mhealth in masses, advocacy, intersectoral collaboration and training of community health workers regarding IYCF nutrition ^23^. Therefore, considering the current situation in Pakistan, we planned to pilot mhealth technology with counseling through LHWs. For this intervention we first conducted a formative study in collaboration with Lady Health Worker (LHW) programme, which helped in the development and implementation of a mhealth based program to counsel women on proper nutritional practices related to infant and young children (IYCF) in a rural periphery located in Islamabad.

Our mhealth intervention was deployed on pregnant and lactating mothers from July 2016 to December 2016 in rural Islamabad. The aim was to test the feasibility and acceptance of mhealth intervention among the target population. A pre-intervention survey was conducted one month prior to the intervention in June 2016 followed by a post intervention survey conducted in May to June 2018 to determine the effectiveness of mhealth intervention in improving infant and young child feeding (IYCF) nutrition related knowledge, attitude and practices among pregnant and lactating mothers in rural Islamabad (Both surveys are available as Extended data ^24^). The specific objective of this post-intervention study was to compare the pre and post mhealth intervention related knowledge, attitude & practices of pregnant and lactating mothers regarding IYCF.

## Methods

### Study design

A quasi-experimental study design was employed to determine the effectiveness of mhealth in improving knowledge, attitude, and practices of pregnant and lactating mothers regarding IYCF in rural Islamabad.

### Study setting

Islamabad is the federal capital territory of Pakistan. According to census conducted in 2017, the total population of Islamabad is more than 2 million ^25^. The rural population of Islamabad comprises of 991,747 individuals with approximately 165,490 number of households ^26^. This study was conducted in Tarlai Kalan which is a rural union council in Islamabad which comprises of around 37,500 households.

### Study population

**Figure 1.**
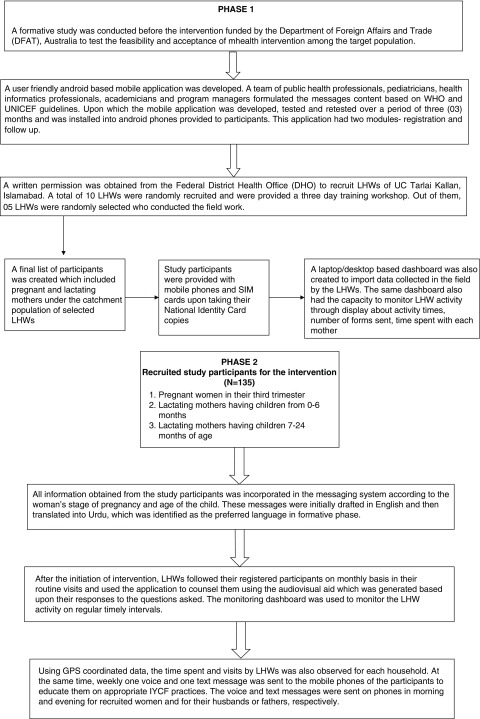
Intervention Phase among pregnant & lactating mothers in rural Islamabad from July to December 2016.

As mentioned, our intervention was deployed to pregnant and lactating women residing in Tarlai, Islamabad (See Figure 1). The study area is covered by the Lady Health Workers (LHWs) who are considered as the first level healthcare providers in this community. Upon availability and approval from the district health office, 10 LHWs were randomly selected and trained on IYCF. Out of these 10 LHWs, 05 were selected on basis of their best performance during the IYCF training. The sampling frame was based on these five selected LHW-wise households where pregnant or lactating mothers residing within the catchment area of Tarlai, Islamabad and have children of ≤2 years of age who were willing to participate in the study were recruited and registered. Their husbands were also invited to participate and after obtaining consent the post-intervention study was conducted. Non-residents, non-consenting cases and mothers with serious co-morbidities were excluded from the study.

### Intervention

A user-friendly audio-visual android based mobile application was developed for LHWs who were then trained and supervised on its use which contained the formulated messages related to IYCF. The content of messages was prepared in English language after extensive review by experts, which were mainly based on WHO and UNICEF guidelines ^14, 27^. It was translated into local language ‘Urdu’ and was then back translated into English language.

***Intervention Design:*** On the basis of results of pre-intervention survey (phase one), it was decided that biweekly voice and text messages on appropriate IYCF practices will be disseminated to the recruited pregnant and lactating mothers as well as the messages will be sent to their mothers in law and husbands. The project team was then trained by a mobile application developer to create their application on the content of voice and text messages to be disseminated.

***Messages:*** A team of public health professionals, paediatricians, health informatics professionals, academicians and program managers formulated the messages based on WHO and UNICEF guidelines. These messages were initially drafted in English, then translated into Urdu and back translated into English. The target audience was pregnant women in their third trimester and lactating mothers of children between 0–24 months of age. The age of child and stage model was employed so that the messages could be sent according to the trimester of the pregnant women or age of the child as the case may be. After these messages were created, they were incorporated into a specialized message scheduling system whereby separate audio and text based message libraries were created to be sent to recipients.

***Mobile Application:*** The application was created over a period of 03 months with testing to optimise the user’s experience. The application had two modules which were registration and follow up. Each module further included two sections, one for the pregnant women from their third trimester and one for children 0–12 months. The questions within each module were drafted in Urdu and included logical checks and errors based on the responses to avoid errors in data entry. The LHWs were to first register pregnant women or mothers of children of 0–12 months of age using the registration form. The included data consisted of name of the mother and the child, age, gender of the child, date of birth, last menstrual period (in case of pregnant women), address, phone number and dietary habits. From the next visit onwards, they were instructed to use the follow up forms to collect monthly data on their dietary intake, supplement intake (in case of pregnant women) and any associated illnesses or problems. A laptop/desktop-based dashboard was also created to import data collected in the field by the LHWs. The same dashboard also had the capacity to monitor the LHW activity through display activity times, number of forms sent, and time spent with each mother. Through the same dashboard the project manager had the capacity to create mobile application users. After creation of the mhealth application, it was installed into android phones and following pretesting by the team members it was then modified.

Once the application was ready and the message library was created, the project team sought written permission from the Federal District Health Office (DHO) to recruit the LHWs of Union Council of Tarlai Kalan for the intervention. The DHO assigned an assistant district coordinator who assisted the project team in recruiting the LHWs. Upon availability of the LHWs, a three-day training workshop was scheduled in a health house. A health house is a household of LHWs. The agenda of the training was to first educate the LHWs on IYCF, explain the objective of the intervention and train them on using the application by providing them application installed smart phones and tablets. A pre- and post-test regarding knowledge on IYCF was also conducted during the training. This was followed by a field visit to test the use of the application in the field. After obtaining the national identity card copies of the selected LHWs, they were then provided with android-based smart phones along with the SIMS and mhealth application installed. Consent to be a part of the study was also obtained for participating in the project, along with their National Identity card copies (this is required by the Pakistan Telecommunication Authority (PTA) for provision of sim cards).

Upon selection, the participating LHWs were then requested to provide a list of eligible pregnant and lactating mothers for the project within their catchment area. These comprised of three groups including all pregnant women in their third trimester, children from 0–6 months of age and those mothers who had children of 7–12 months of age. Before including their names in the study, the LHWs were advised to describe the purpose of the mhealth project to the respondents or the caretakers (which were assumed as those individuals who were responsible for the care of infant or the child at their homes). Only those individuals or caretakers who had consented to be a part of the intervention were included. On average, around 10–15 individuals’ names were provided under each group by each LHW. Once the lists were provided, three individuals under each group for each LHW were selected and assigned to be included in the intervention through a lottery / draw method. These respondents were randomly picked out of all the recruited participants whose names were included in a draw box to receive the mhealth intervention. The participants consisted of primary participants who were pregnant women and mothers of children of 0–12 months of age followed by secondary participants who were mothers in law/grandmothers and husbands/fathers of the primary participants. The purpose of including the other family members was sociocultural. As in the first phase of the project; it was indicated that for a successful delivery of mhealth intervention, involving husbands and mothers in law will be very important. This was so that the participants would own the intervention and consider themselves as participants in the study.

Once the final list of participants was created, a one-day inaugural session was organized for them near to their place of their residence so as to brief them about the intervention. A pre-intervention survey was conducted after which they were provided with mobile phones along with SIM cards upon obtaining their National Identity Card copies and their written consent for participating in the study. Once all the information was obtained from the participants, it was then incorporated in the messaging system according to the stage of pregnancy and age of the child for the purpose of dissemination of IYCF nutrition related knowledge. From the next day, mhealth intervention was initiated as all the LHWs registered their participants and they were then followed up every month in their routine visits. They were also counseled and provided with information on using the mobile application and an audio-visual aid which was generated based upon their responses to the questions being asked regarding IYCF.

At the same time, a weekly voice and text message was sent to the mobile phones of the participant to educate them on appropriate IYCF practices. The voice message was sent on Tuesday mornings to the phones of the females and evenings for the husbands/fathers, whereas the text message was sent on Thursday at the same time. The content of both the voice and text messages was the same to avoid confusion. Every month the pregnant women and mothers of children were also called up through our call centre to inquire about the routine LHW visit, whereby they were asked about the visit and whether if they had received the voice and text messages. They were also asked about the content of sent messages which they had received. A monitoring dashboard was used to monitor the LHW activity, whereby the project manager observed whether the visits were actually made using GPS coordinated data. Similarly, the time when the visit was made was also noted along with the time spent in each household. This intervention lasted for six months starting from July to December 2016 followed by a short post intervention research consisting of a focus group discussion with the mothers.

### Data collection instrument

A structured, post intervention questionnaire was used for data collection which was developed on the same lines as that of the pre-intervention survey. The initial version of questionnaire was developed through extensive review of literature and experts’ review ^20, 27^. The questionnaire’s Part A comprised of socio-demographic characteristics of the study participants which included age and education level of the women, number of children, family size, place of birth, and mode of delivery. Part B and C contained questions related to breastfeeding, exclusive breastfeeding, and complementary feeding.

The study variables related to knowledge, attitude and practices regarding IYCF were timely initiation of breastfeeding after birth, advisable duration of breastfeeding and exclusive breastfeeding, complementary feeding initiation and continuation, and practices related to prelacteal feeding. The data was collected through telephonic interviews which were indicated as a preference in our formative study. Only if the woman was unreachable via the phone were they then visited at their house for the interview which was facilitated by the respective LHW of the respondents’ catchment area.

### Statistics analysis

The collected data was double-entered in EpiData software version 3.1. It was analyzed using SPSS version 21. The total sample size was 135 eligible women which were recruited based on the sampling frame created earlier for the intervention phase. Both descriptive and inferential statistics are reported in frequencies and percentages, including the percentage difference for pre and post knowledge, attitude, and practices related to IYCF nutrition.

### Ethical consideration

Ethical clearance was obtained from Hospital Ethics Committee of Pakistan Institute of Medical Sciences (PIMS), Islamabad ^28^. Informed consent (both written and verbal) was obtained from all study participants prior to their recruitment in the study. These women and their husbands were approached and were explained about the study purpose. Their queries to the study were addressed and they were provided with necessary information to contact in case of withdrawing from the study. They were also ensured about their privacy and confidentiality to be protected.

## Results

Table 1 shows baseline characteristics of the 135 mothers, out of which 49 (36.3%) women belong to the age group of 25 to 29 years of age. The mean age of these pregnant and lactating mothers was 30.5 years ± 4.5 SD. Out of 135 women, 71 (52.6%) had 3 children and on average had 7 family members. Most women i.e. 59 (43.7%) had their education up to Matriculation and Intermediate followed by primary level of education for 48 (35.6%) of the women. The occupation status of 128 (94.8%) of these women was ‘unemployed’. The birth place of children as reported by 77 (57.3%) of women was a government facility, and the mode of delivery of 86 (63.7%) women was reported as ‘normal’. The common source of water was ‘tap water’ in the households according to 50 (37%) of women.

**Table 1.** Socio-Demographic characteristics of pregnant & lactating mothers in rural Islamabad, during 2016–2018 (n=135).

Characteristics	Frequency	Percentage (%)
**Age in years (30.5± 4.5)**		
15–19	01	0.7
20–24	10	7.4
25–29	49	36.3
30–34	44	32.6
35–39	31	23.0
**Number of Children (alive)**		
01	05	3.7
02	32	23.7
03	71	52.6
04	14	10.4
05	13	9.6
**Family Size**		
Average	6.56	-
Minimum	03	2.2
Maximum	13	0.7
**Education**		
Illiterate *	13	9.6
Primary	48	35.6
Matric/Intermediate	59	43.7
Graduation	15	11.1
**Occupation**		
Employed	07	5.2
Unemployed	128	94.8
**Place of Birth**		
Government Facility	77	57.3
Private Facility	58	43.0
**Mode of Delivery**		
Normal Delivery	86	63.7
C-Section	49	36.3
**Monthly LHW Visits**		
Once	55	40.7
Twice or more	49	36.3
Never	22	16.3
Don’t Know/Don’t Remember	09	6.7
**Source of Water**		
Tap Water	50	37.0
Well Water	46	34.1
Boiled Water	16	11.9
Mineral Water	08	5.9
Tube Well	15	11.9

* No Formal Education, LHW=Lady Health Worker

The overall pre-intervention knowledge of 34 pregnant mothers regarding breastfeeding, exclusive breastfeeding and complementary feeding was 75.6%which decreased to 46.7% among 21of these women even after the mhealth intervention. This emphasizes the need of increasing awareness among pregnant women, in particular ( Table 2). Overall, findings of our survey elucidated that the mhealth intervention was effective in improving the overall knowledge of mothers regarding IYCF from 74 (54.8%) in 2016 to 94 (69.6%) in 2018 after the intervention ( Table 4)

**Table 2.** Knowledge regarding infant and young child feeding (IYCF) nutrition among pregnant & lactating women in rural Islamabad, during 2016–2018 (n=135).

Knowledge Questions	Correct Responses	Before (%)	After (%)	Percentage Difference
**Breastfeeding initiation after birth**	Within one hour of birth	80.2	74.8	-5.4
**Prelacteal feed for baby**	Harmful	38.5	66.7	28.2
**Benefits of Colostrum**	Rich in nutrients & provides immunity	58.5	81.5	23
**Advisable duration of** **Breastfeeding**	1–2 year	94.5	81.5	-13.0
**Understanding about exclusive** **breastfeeding**	Exclusively giving mother’s milk for first 6 months and nothing else	80.2	79.3	-0.9
**Complementary food initiation**	At 7 months	7.7	5.9	-1.8

The overall attitude regarding IYCF among 59 (43.7%) of the mothers before intervention, and among 86 (63.7%) of the mothers after intervention, was found to be positive. Whereas; overall practices of 22 (16.3%) mothers before intervention and 88 (65.2%) of the mothers after intervention were found adherent to good practices ( Table 3). A noticeable percentage increase in knowledge related to prelacteal feeding considered as harmful and the benefits of colostrum was 28.2% and 23%, respectively ( Table 2).

**Table 3.** Overall knowledge, attitude & practices regarding infant and young child feeding (IYCF) nutrition among pregnant & lactating women in rural Islamabad, during 2016–2018 (n=135).

	Pre-Intervention (2016)		Post-Intervention (2018)	
	N	(%)	N	(%)
**Knowledge**	74	54.8%	94	69.6%
**Attitude**	59	43.7%	86	63.7%
**Practice**	22	16.3%	88	65.2%

**Table 4.** Attitude & practices regarding infant and young child feeding (IYCF) nutrition among pregnant & lactating women in rural Islamabad, during 2016–2018 (n=135).

Attitude Questions	Correct Response	Before (%)	After (%)	Percentage Difference
**Prelacteal feed be given to born baby**	No	62.9	63.7	0.8
**A mother should breastfeed when she is ill**	Should breastfed	73.6	78.5	4.9
**Consistency of complementary food**	Thick and sticky	12.1	58.5	46.4
**Practice Questions**				
**Breastfeed baby within 1 hour of birth**	Yes	51.6	74.8	23.2
**Complementary feeding**	3 Times	41.8	40.7	-1.1
**Additional food during first 6 months**	Nothing	0.0	66.7	66.7

A percentage difference of 46.4 was observed in attitude of mothers towards consistency of food consumed by their children, which was 12.1% before intervention, and was found to be adequate among 58.5% of the mothers after intervention. Furthermore, practices regarding complementary feeding and additional foods during the first six months of infancy were 0.0% before the intervention which was significantly raised to 66.7% among these mothers ( Table 4). In addition, 55 (40.7%) of the mothers reported to be visited ‘once’ by LHW, followed by 49 (36.3%) of the mothers who were visited ‘twice’ on a monthly basis ( Table 1). Pre- and post-intervention findings are available as Underlying data ^24^.

## Discussion

For child survival, growth and development, a key strategy is to improve IYCF related practices which is becoming an essential component of child health programs in various countries ^29^. The results of our post-mhealth intervention survey regarding IYCF conducted in a rural territory in Islamabad yielded to be effective in improving the knowledge, attitude, and practices of pregnant and lactating mothers. Based on findings of our earlier research conducted on the same study population we found that community-based nutritional intervention such as ‘mhealth’ offer new opportunities for effective and efficient service delivery, resource utilization, and improving access to healthcare ^30^.

Improving IYCF practices in poor resource setting can be effectively contextualized through information technology involving mhealth. Specific socio-cultural and socio-economic barriers hindering access of mothers for acquiring information related to IYCF must be overcome in order to reduce the prevailing burden of preventable malnutrition ^31^. Studies suggest that maternal literacy plays important role whereas healthcare services can be augmented through the use of mobile phone-based technology such as mhealth ^31, 32^. It offers enormous opportunities for improving health indicators related to maternal, new born and child health specifically in rural settings. It was found in one study that mhealth or SMS-based health education could provide an essential chance to educate pregnant and lactating mothers about antenatal care (ANC) visits, child birth, and education related to family planning ^32^. This indicates that there is a potential capacity to implement mhealth based IYCF which may render opportunities for scaling up the intervention in rural Islamabad. The findings of our survey elucidate that specific focus should be placed on the components of knowledge related to breastfeeding and exclusive breastfeeding during early infancy. However; relevance and quality of mhealth to other components of maternal and child health must be rigorously studied to promote the proliferation of mobile phones as a source of acquiring health information in LMICs.

Despite the improvement in overall knowledge, attitude and practices of women related to IYCF in our study, certain important components related to breastfeeding showed steady findings. The knowledge of women regarding advisable duration of breastfeeding, early initiation of breastfeeding after birth, and timely complementary feeding initiation with additional food to be given to in early 6 months of infancy showed no significant change after mhealth intervention. This could be attributed to a prolong washout period after the deployment of mhealth intervention among the mothers or it can possibly subject to recall bias. Despite this more than a quarter of women in our study still practised and considered prelacteal feed such as honey and water as advantageous for the infant. This was found to be consistent to the findings of research studies conducted in Myanmar, Ethopia & India where prelacteal feeding was perceived as a cultural practice and was related to maternal beliefs ^33– 36^.

There seems to be a paucity of relevant available literature on assessing the effectiveness of mhealth, particularly in the context of infant and young child nutrition particularly in Pakistan ^30^, which signifies its importance in implementing such interventions in poor resource settings. In contrast to our earlier research findings on testing the acceptance of mhealth among women residing in Tarlai Kalan Islamabad, a study from Sri Lanka demonstrated that women preferred to interact with healthcare providers on their maternity and child health needs ^37^. Contrary to it, in our study majority of women favoured the use of mobile phones to access information related to infant and young child feeding. On the basis of which, we therefore recommend scaling up of the health intervention in poor resource settings for the purpose of providing knowledge and increasing awareness regarding IYCF. Our study findings reflect that extensive mobile coverage has emerged as an innovative tool in rural Islamabad, and has acted as a facilitator which can effectively reach the underserved communities for providing health as well as education regarding infant and young child nutrition.

### Strengths and limitations

• Overall, the strength of the deployed intervention lies in an increase in the practices of mothers related to IYCF nutrition

• This was a novel intervention, the first of its kind in Pakistan

• We have managed to incorporate the intervention within the existing LHW program rather integrating a new intervention so as to enable the intervention to be scaled up feasibly

• One of our study limitations is that we conducted telephonic interviews which can introduce potential biases in responses of the mothers unlike in face-face interview approach.

• Quasi-experimental designs have limitations as compared to experimental designs since it includes no randomization

## Conclusion

Our study indicates that community-based nutritional interventions using mhealth are innovative and effective in increasing IYCF related knowledge, attitude and practices among mothers. Cost-effectiveness of such behaviour change approaches and interventions should be assessed for future implementation in maternal and child health related programmes. Further experimental studies must be explored to validate the findings in Pakistani context.

## Software availability

The source code of android phone-based application developed for the Lady Health Workers (LHWs) under the project “Sehatmnd Kl” is the property of Maternal, Neonatal and Child Health Research Network (MNCHRN) and cannot be made public.

All content used in the app to provide information to the recruited mothers is available as Extended data ^24^.

## Data availability

### Underlying data

Open Science Framework: Effectiveness of mhealth on IYCF. https://doi.org/10.17605/OSF.IO/VRHA5 ^24^

This project contains the following underlying data:

- Data Set Epi Data.zip (Data entry sheet on Epi Data 3.1)
- Post Analysis.sav (Output file of data analysis on SPSS version 21)
- Pre & Post Scoring _Pregnant.sav (SPSS file of pre & post entered data)

### Extended data

Open Science Framework: Effectiveness of mhealth on IYCF. https://doi.org/10.17605/OSF.IO/VRHA5 ^24^

This project contains the following extended data:

- Finalized__0-6__months[1].pdf (Pre-intervention survey for 0–6 month infants)
- Finalized___7-12_months[1].pdf (Pre-intervention survey for 0–6 month infants)
- Finalized_Pregnancy_survey[1].pdf (Pre-intervention survey for mothers in final trimester)
- Intervention Application.zip (content from Android app)

## References

[1] Ahmed T Hossain M Sanin KI: Global burden of maternal and child undernutrition and micronutrient deficiencies. *Ann Nutr Metab.* 2012;61 Suppl 1:8–17. 10.1159/000345165 23343943

[2] Weise AS: Global Nutrition Targets 2025: Stunting policy brief. World Health Organization;2014 Reference Source

[3] Prado EL Dewey KG: Nutrition and brain development in early life. *Nutr Rev.* 2014;72(4):267–84. 10.1111/nure.12102 24684384

[4] Nelson CA Luciana ML: Handbook of developmental cognitive neuroscience. MIT Press;2008;923 Reference Source

[5] Wiggins RC Fuller G Enna SJ: Undernutrition and the development of brain neurotransmitter systems. *Life Sci.* 1984;35(21):2085–94. 10.1016/0024-3205(84)90507-1 6149444

[6] Infant and young child feeding. Reference Source

[7] Alam M D’Este C Banwell C: The impact of mobile phone based messages on maternal and child healthcare behaviour: a retrospective cross-sectional survey in Bangladesh. *BMC Health Serv Res.* 2017;17(1):434. 10.1186/s12913-017-2361-6 28645278 PMC5482970

[8] Biks GA Tariku A Wassie MM: Mother's Infant and Young Child Feeding (IYCF) knowledge improved timely initiation of complementary feeding of children aged 6-24 months in the rural population of northwest Ethiopia. *BMC Res Notes.* 2018;11(1):593. 10.1186/s13104-018-3703-0 30115114 PMC6097428

[9] Latif S Rana R Qadir J: Mobile Health in the Developing World: Review of Literature and Lessons from a Case Study. *IEEE Access.* 2017;5:11540–56. 10.1109/ACCESS.2017.2710800

[10] NIPS: Pakistan Demographic and Health Survey 2017-18 Key Indicators Report.2018 Reference Source

[11] National Institute of Population Studies (NIPS) [Pakistan] and ICF International: Pakistan Demographic and Health Survey 2012-13. Islamabad, Pakistan, and Calverton, Maryland, USA: NIPS and ICF International.2013 Reference Source

[12] Manikam L Sharmila A Dharmaratnam A: Systematic review of infant and young child complementary feeding practices in South Asian families: the Pakistan perspective. *Public Health Nutr.* 2018;21(4):655–68. 10.1017/S1368980017002956 29151370 PMC5851056

[13] World Health Organization, UNICEF: Infant and young child feeding counselling: an integrated course. Who.2006;1–265. Reference Source

[14] World Health Organization (WHO): Global Strategy for Infant and Young Child Feeding. World Health Organization;2003 Reference Source

[15] Hafeez A Mohamud BK Shiekh MR: Lady health workers programme in Pakistan: challenges, achievements and the way forward. *J Pak Med Assoc.* 2011;61(3):210–5. 21465929

[16] Jalal S: The lady health worker program in Pakistan--a commentary. *Eur J Public Health.* 2011;21(2):143–4. 10.1093/eurpub/ckq199 21278131

[17] USAID: mHealth Compendium.2012 Reference Source

[18] Alam M Khanam T Khan R: Assessing the scope for use of mobile based solution to improve maternal and child health in Bangladesh: A case study. Proc 4th ACM/IEEE Int Conf Inf Commun Technol Dev.2010; 3. Reference Source

[19] Mobile Solutions Aiding Knowledge For Health Improvement– Lata Medical Research Foundation (Reg. No. E-1559, Nagpur, India).2017 Reference Source

[20] World Health Organization: WHO | Global Strategy for Infant and Young Child Feeding.WHO. World Health Organization;2010 Reference Source

[21] Khan NUZ Rasheed S Sharmin T: How can mobile phones be used to improve nutrition service delivery in rural Bangladesh? *BMC Health Serv Res.* 2018;18(1):530. 10.1186/s12913-018-3351-z 29986733 PMC6038298

[22] Avula R Oddo VM Kadiyala S: Scaling-up interventions to improve infant and young child feeding in India: What will it take? *Matern Child Nutr.* 2017;13 Suppl 2:e12414. 10.1111/mcn.12414 29032618 PMC6866129

[23] Mahmood H Suleman Y Hazir T: Overview of the infant and young child feeding policy environment in Pakistan: Federal, Sindh and Punjab context. *BMC Public Health.* 2017;17(Suppl 2):474. 10.1186/s12889-017-4341-5 28675134 PMC5496022

[24] Khan SA Mahmood H Fatima R: Effectiveness of mhealth on IYCF.2019 10.17605/OSF.IO/VRHA5

[25] PROVINCE WISE PROVISIONAL RESULTS OF CENSUS - 2017. Reference Source

[26] POPULATION ADMIN UNIT NO OF HH POPULATION AND HOUSEHOLD CENSUS 2018. Reference Source

[27] UNICEF: Programming Guide Infant and Young Child Feeding.Nutr Sect UNICEF.2011;173 Reference Source

[28] Pakistan Institute of Medical Sciences - PIMS. Reference Source

[29] Save the Children UK: Strengthening Infant and Young Child Feeding Programming and Planning for Emergency Preparedness and Response. *Proceedings of an international workshop* Organised and funded by Save the Children UK in co-operation with UNICEF’s IYCN and Emergencies Units. Reference Source

[30] Mildon A Sellen D: Behaviour Change Communication Using Mobile Phones: Implications for Infant and Young Child Feeding Interventions. *FASEB J.* Reference Source

[31] Khan GN Ariff S Khan U: Determinants of infant and young child feeding practices by mothers in two rural districts of Sindh, Pakistan: a cross-sectional survey. *Int Breastfeed J.* 2017;12:40. 10.1186/s13006-017-0131-z 28936229 PMC5603092

[32] Uddin J Biswas T Adhikary G: Impact of mobile phone-based technology to improve health, population and nutrition services in Rural Bangladesh: a study protocol. *BMC Med Inform Decis Mak.* 2017;17(1):101. 10.1186/s12911-017-0502-9 28683742 PMC5500967

[33] Hmone MP Dibley MJ Li M: A formative study to inform mHealth based randomized controlled trial intervention to promote exclusive breastfeeding practices in Myanmar: incorporating qualitative study findings. *BMC Med Inform Decis Mak.* 2016;16(1):60. 10.1186/s12911-016-0301-8 27260252 PMC4893226

[34] Mane SS Chundi PR: Infant and young child feeding practices among mothers in Hyderabad, Telangana. *Int J Community Med Public Heal.* 2017;4(10):3808 10.18203/2394-6040.ijcmph20174255

[35] Sriram S Soni P Thanvi R: NATIONAL JOURNAL OF MEDICAL RESEARCH KNOWLEDGE, ATTITUDE AND PRACTICES OF MOTHERS REGARDING INFANT FEEDING PRACTICES.2013;3(2):147–150. Reference Source

[36] Tekaly G Kassa M Belete T: Pre-lacteal feeding practice and associated factors among mothers having children less than two years of age in Aksum town, Tigray, Ethiopia, 2017: a cross-sectional study. *BMC Pediatr.* 2018;18(1):310. 10.1186/s12887-018-1284-7 30253771 PMC6156946

[37] Weerasinghe MC Senerath U Godakandage S: Use of Mobile Phones for Infant and Young Child Feeding Counseling in Sri Lankan Tea Estates: A Formative Study. *Qual Rep.* 2016;21(5). Reference Source

