# Supplementary material for: Effectiveness of a mobile health intervention on infant and young child feeding among children ≤ 24 months of age in rural Islamabad over six months duration
Source: F1000Res. 2019 Oct 14;8:551. Originally published 2019 Apr 25. [Version 3] doi: 10.12688/f1000research.17037.3 (PMC6820820; doi:10.12688/f1000research.17037.3)
Supplement: Peer review history

# Peer review: Reviewer response for version 3

**DOI:** 10.5256/f1000research.23029.r55098

**Published:** 2019-10-28

**Authors:** Alexandre Delamou

**Affiliations:** 1 Department of Public Health, Gamal Abdel Nasser University of Conakry, Conakry, Guinea

I thank the authors for their efforts to address all my comments in their new version. I have no additional comments.

I confirm that I have read this submission and believe that I have an appropriate level of expertise to confirm that it is of an acceptable scientific standard.

# Peer review: Reviewer response for version 2

**DOI:** 10.5256/f1000research.22004.r51735

**Published:** 2019-08-19

**Authors:** Zohra Lassi

**Affiliations:** 1 The Robinson Research Institute, The University of Adelaide, Adelaide, SA, Australia

I feel the reference should be cited at the end of the sentence. My comment #2 and #5 needs to be addressed.

I confirm that I have read this submission and believe that I have an appropriate level of expertise to confirm that it is of an acceptable scientific standard, however I have significant reservations, as outlined above.

# Response

**Published:** 2019-10-08

**Authors:** Subhana Khan Akber

**Affiliations:** Islamabad, Pakistan, Pakistan

References have been cited at the end of the sentences mentioned in comment number 2 and 5.

# Response

**Published:** 2019-10-08

**Authors:** Subhana Khan Akber

**Affiliations:** Islamabad, Pakistan, Pakistan

References have been updated.

# Peer review: Reviewer response for version 2

**DOI:** 10.5256/f1000research.22004.r51736

**Published:** 2019-07-29

**Authors:** Alexandre Delamou

**Affiliations:** 1 Department of Public Health, Gamal Abdel Nasser University of Conakry, Conakry, Guinea

I thank the authors for their efforts to address the comments made on the first version. Most of my comments have been addressed but still, I think they omitted one:

1. In the limitations section, it is important to acknowledge the fact that the quasi-experimental design bears some limitations as compared to experimental designs. In the conclusion section, I would say that we need experimental designs to confirm/validate their results in the Pakistani context.

Thank you.

I confirm that I have read this submission and believe that I have an appropriate level of expertise to confirm that it is of an acceptable scientific standard, however I have significant reservations, as outlined above.

# Response

**Published:** 2019-10-08

**Authors:** Subhana Khan Akber

**Affiliations:** Islamabad, Pakistan, Pakistan

Revised as per kind suggestion.

# Response

**Published:** 2019-10-08

**Authors:** Subhana Khan Akber

**Affiliations:** Islamabad, Pakistan, Pakistan

Revised as per kind suggestions.

# Peer review: Reviewer response for version 1

**DOI:** 10.5256/f1000research.18626.r47681

**Published:** 2019-05-23

**Authors:** Alexandre Delamou

**Affiliations:** 1 Department of Public Health, Gamal Abdel Nasser University of Conakry, Conakry, Guinea

I have provided my comments directly in a PDF document because the manuscript pages are not numbered to facilitate the reviewer's work.

 My main comments are:

- The intent of the authors is good. They made a lot of effort to well describe the intervention and conduct pre- and post-intervention surveys.

However:

- The design does not optimistic conclusions. I think the authors should be very modest and discuss some key limitations: the selection was not randomly done, there was no comparator to your study (cluster randomized trial). Also, the questionnaire was quantitative and you did not capture the qualitative aspect of the intervention (feasibility, acceptability, sustainability, etc).
- The introduction is long but the authors did not use the space to justify the study in Pakistan. Just saying that "there is insufficient information" is not the good justification.
- There are many typos in the text, I have flagged some but the text needs some corrections.

I confirm that I have read this submission and believe that I have an appropriate level of expertise to confirm that it is of an acceptable scientific standard, however I have significant reservations, as outlined above.

# Response

**Published:** 2019-07-21

**Authors:** Subhana Khan Akber

**Affiliations:** Islamabad, Pakistan, Pakistan

**Reviewer 3:** Alexandre Delamou, Department of Public Health, Gamal Abdel Nasser University of Conakry, Conakry, Guinea

 I have provided my comments directly in a PDF document because the manuscript pages are not numbered to facilitate the reviewer's work.

**Suggested changes revised in the manuscript. PDF file is sent for review comments.**

 My main comments are:

 The intent of the authors is good. They made a lot of effort to well describe the intervention and conduct pre- and post-intervention surveys.

 However: The design does not optimistic conclusions. I think the authors should be very modest and discuss some key limitations: the selection was not randomly done, there was no comparator to your study (cluster randomized trial). Also, the questionnaire was quantitative and you did not capture the qualitative aspect of the intervention (feasibility, acceptability, sustainability, etc).

**We opted for quasi-experimental (pre-post) design and convenience-based sampling was done for the selection of participants form the sampling frame. The qualitative component, we covered in the formative study where we tested the acceptance of the intervention mentioned in the introduction section, last paragraph.**

 The introduction is long but the authors did not use the space to justify the study in Pakistan. Just saying that "there is insufficient information" is not the good justification.

**Revised in the introduction.**

 There are many typos in the text, I have flagged some but the text needs some corrections.

**Corrections have been made in the text.**

# Peer review: Reviewer response for version 1

**DOI:** 10.5256/f1000research.18626.r47677

**Published:** 2019-05-21

**Authors:** Ejaz Ahmad Khan

**Affiliations:** 1 Department of Epidemiology and Biostatistics, Health Services Academy (HSA), Islamabad, Pakistan

General comments:

 This is a good effort by the authors to introduce mHealth in overcoming the barriers to achieving optimum IYCF. The article is not written very well. There are numerous English language comprehension and spelling (perhaps typo) mistakes. The authors leave the reader confused about their sampling frame at several places, and text is some times extremely haphazard. The tables are cited irrespective of their order as they appear in the article. Discussion lacks critical points from results discussed. There is an element of publication bias and misinterpretation of technical terms such as Recall Bias (contrary to how it is defined in text books of epidemiology). About 30% of the references are more than five years old. 

 Specific comments:

 Abstract:

- The abstract reports only the positive findings without highlighting the negative findings. It also concludes for effectiveness, whereas, no test of significance was applied, and just uses the difference in percentages as proof of significant effectiveness. 

Methods:

- Ten can be written as 10, as numbers below 10 are written in words only.
- For intervention part, the authors had messages translated to Urdu from English just once. However, the standard is to back translate it in English so that they are exactly the same as they were before the initial translation. 
- The paragraph on "intervention design" needs rephrasing as the text is confusing. Similarly, the paragraph on "messages" also needs rephrasing, so that the reader easily understand what the authors wanted them to know.
- Sampling frame creates big confusion. In the start it is 0-24 months of age children. But then it changes to 0-12 months in the later text (mobile application part). Further, in the flow diagram, this age group is further subdivided into 0-6 and 7-24 months of age. It is unclear how the application designed only for 0-12 months of age could have catered for age group beyond 12 months. The flow diagram has repeatedly wrong spellings of "participants". In paragraph three, following the heading "mobile application" again contradicts the 7-24 months of age and states 7-12 months of age.
- age 6, paragraph two, the text has not been able to explain to the reader whether the SIM and mobile were acquired for the LHWs or for all the participants. This should be clearly stated and differentiated. 

Additional points for methods:

- For practices, why were there non-direct or indirect observations? The acquired knowledge of the participants during the intervention can lead to over reporting of good practices to please the researchers (Hawthorne effect). Furthermore, did the researchers take into an account the effect of other sources of knowledge (confounders), which could supplement or negatively impact their intervention, and how did they take care of them and controlled those factors?

Results:

- Results are divided into descriptive and inferential statistics. However, no test of significance was applied for the inferential statistics. Just on the bases of difference of percentages in pre- and post- is insufficient to claim significant effectiveness.
- The results should be written in order of the objectives of the study as they were achieved.
- The tables are cited without proper and ascending sequence. 

Discussion:

- Instead of discussing the mHealth intervention in length, depicting their inclination towards supporting their positive results only, the authors should do impartial and unbiased discussion. They should also discuss the implication of negative results, why some practices also increased while a few declined, what could be the underlying factors (supported by literature), claiming that the women favored the use of mobile phone to access information was not supported by any result given in the text.

References:

- About 30% of references were of more than five years old.

I confirm that I have read this submission and believe that I have an appropriate level of expertise to confirm that it is of an acceptable scientific standard, however I have significant reservations, as outlined above.

# Response

**Published:** 2019-07-21

**Authors:** Subhana Khan Akber

**Affiliations:** Islamabad, Pakistan, Pakistan

**Reviewer 2:** Ejaz Ahmad Khan, Department of Epidemiology and Biostatistics, Health Services Academy (HSA), Islamabad, Pakistan

 General comments:

 This is a good effort by the authors to introduce mHealth in overcoming the barriers to achieving optimum IYCF. The article is not written very well. There are numerous English language comprehension and spelling (perhaps typo) mistakes. The authors leave the reader confused about their sampling frame at several places, and text is sometimes extremely haphazard. The tables are cited irrespective of their order as they appear in the article. Discussion lacks critical points from results discussed. There is an element of publication bias and misinterpretation of technical terms such as Recall Bias (contrary to how it is defined in text books of epidemiology). About 30% of the references are more than five years old.

**The comprehension mistakes have been corrected. Sentences regarding sampling frame have been rephrased. Tables have been cited correctly and in ascending order. We mentioned that we were handicapped on finding useful literature related to mhealth in our context for the discussion section. Recall bias is a systematic error introduced in the study due to providing inaccurate answers to questions, similarly mothers who were interviewed were found indecisive to answer the questions at some points which can be attributed to long wash out period after intervention. There are 12 references that are more than five years old, due to limited available data so we searched literature that is available in past 10 years.**

 Specific comments:

 Abstract:

 The abstract reports only the positive findings without highlighting the negative findings. It also concludes for effectiveness, whereas, no test of significance was applied, and just uses the difference in percentages as proof of significant effectiveness.

**Negative findings have been reported in the results and tables. Since it was not a trial therefore by effectiveness we mean the success of deploying mhealth intervention for IYCF. Hence, differences were calculated pre-post to assess the increase in knowledge, attitude and practices among the study participants.**

 Methods:

 Ten can be written as 10, as numbers below 10 are written in words only.

**Revised**

 For intervention part, the authors had messages translated to Urdu from English just once. However, the standard is to back translate it in English so that they are exactly the same as they were before the initial translation.

**The messages were back translated into English language, the text has been revised.**

 The paragraph on "intervention design" needs rephrasing as the text is confusing. Similarly, the paragraph on "messages" also needs rephrasing, so that the reader easily understand what the authors wanted them to know.

**Rephrased**

 Sampling frame creates big confusion. In the start it is 0-24 months of age children. But then it changes to 0-12 months in the later text (mobile application part). Further, in the flow diagram, this age group is further subdivided into 0-6 and 7-24 months of age. It is unclear how the application designed only for 0-12 months of age could have catered for age group beyond 12 months. The flow diagram has repeatedly wrong spellings of "participants". In paragraph three, following the heading "mobile application" again contradicts the 7-24 months of age and states 7-12 months of age.

**The mobile application part is the intervention phase where mothers having children of 0-12 months of age were recruited in 2016 but messages were sent according to the age groups defined which were from 0-6 months and 7-12 months. Later on in the post-intervention survey which was conducted in 2018, we included the same population but the age of the children was set 0-24 months so to match the ages of children. Spelling errors have been corrected. In the paragraph three, the age group refers to the pre-intervention phase.**

 Age 6, paragraph two, the text has not been able to explain to the reader whether the SIM and mobile were acquired for the LHWs or for all the participants. This should be clearly stated and differentiated.

**Revised. The text has been mentioned in paragraph two, line number 11.**

 Additional points for methods:

 For practices, why were there non-direct or indirect observations? The acquired knowledge of the participants during the intervention can lead to over reporting of good practices to please the researchers (Hawthorne effect). Furthermore, did the researchers take into an account the effect of other sources of knowledge (confounders), which could supplement or negatively impact their intervention, and how did they take care of them and controlled those factors?

**Practice-based knowledge questions were asked from the study participants without observation. We did not took into account Hawthorne effect or other confounding factors though we have mentioned that this evaluation was conducted after 2016 in 2018 and have mentioned in limitations about potential biases.**

 Results:

 Results are divided into descriptive and inferential statistics. However, no test of significance was applied for the inferential statistics. Just on the bases of difference of percentages in pre- and post- is insufficient to claim significant effectiveness.

**For effectiveness we referred to it the success of using mhealth for dissemination of knowledge regarding IYCF.**

 The results should be written in order of the objectives of the study as they were achieved.

 The tables are cited without proper and ascending sequence.

**Revised and table have cited correctly in ascending order.**

 Discussion:

 Instead of discussing the mHealth intervention in length, depicting their inclination towards supporting their positive results only, the authors should do impartial and unbiased discussion. They should also discuss the implication of negative results, why some practices also increased while a few declined, what could be the underlying factors (supported by literature), claiming that the women favored the use of mobile phone to access information was not supported by any result given in the text.

**Decrease of some practices has been ascribed to prolong wash period mentioned in the text paragraph 3 line number 6. The discussion might be read as narrow due to limited evidence on use of mhealth for IYCF knowledge and quality of mhealth for MNCH programs. But negative results have been discussed and supported by literature in paragraph two line numbers 3 & 5, paragraph three line number 10. The use of mobile phone to access information was been demonstrated by the results of our formative study.**

 References:

 About 30% of references were of more than five years old.

**There are 12 references that are more than five years old, due to limited available data so we searched literature that is available in past 10 years.**

# Peer review: Reviewer response for version 1

**DOI:** 10.5256/f1000research.18626.r47680

**Published:** 2019-05-08

**Authors:** Zohra Lassi

**Affiliations:** 1 The Robinson Research Institute, The University of Adelaide, Adelaide, SA, Australia

**Comments:**

- Abstract-Results: After the intervention, the overall knowledge of mothers regarding IYCF nutrition was raised to 69.6% among 94 mothers as compared to 74 (54.8%). Please complete the sentence, 74 (54.8%) in the comparison group? 
- Introduction - Second Paragraph - first line: Needs a reference. 
- Introduction - Second Paragraph - third line: Reference 6 should be hyper-script. 
- Introduction - Second Paragraph: Developing countries should be replaced with low- and middle-income countries. 
- Introduction - Second Paragraph: The sentence "According to the Pakistan Demographic Health Survey (PDHS, 2018), almost 38% of children under five years of age are stunted, 23% are underweight, and 7% are wasted" needs a reference. 
- The words developing countries and LMICs should be used consistently. In the current form, both have been used interchangeably. 
- The IYCF acronym was introduced in the first paragraph of the Introduction, and still used in full several times in the Introduction. 
- Abstract - methods: The date of study mentioned was May to June 2018. However in the Introduction, it was mentioned that it was piloted in 2016 and evaluated between April to June 2018. Please check the dates and provide the correct one. 
- Under mobile applications - Second Paragraph: Please add "study" in the sentence: "Consent to be a part of the was also obtained for participating in the project,"
- Please provide the name of the smart phone application that was created? 
- It would be more informative, if the interface of the application is attached to the paper and the type of options available for the participants to receive information on IYCF. 
- Discussion: Please indicate if there is agreement of the results with the earlier studies? 

I confirm that I have read this submission and believe that I have an appropriate level of expertise to confirm that it is of an acceptable scientific standard, however I have significant reservations, as outlined above.

# Response

**Published:** 2019-07-21

**Authors:** Subhana Khan Akber

**Affiliations:** Islamabad, Pakistan, Pakistan

**Responses to Reviewers**

**Reviewer 1:** Zohra Lassi, The Robinson Research Institute, The University of Adelaide, Adelaide, SA, Australia

- Abstract-Results: After the intervention, the overall knowledge of mothers regarding IYCF nutrition was raised to 69.6% among 94 mothers as compared to 74 (54.8%). Please complete the sentence, 74 (54.8%) in the comparison group?

**Added as suggested**

- Introduction - Second Paragraph - first line: Needs a reference.

**Provided at the end of completing the sentences at line 4**

  

- Introduction - Second Paragraph - third line: Reference 6 should be hyper-script.

 
**Revised as suggested**

- Introduction - Second Paragraph: Developing countries should be replaced with low- and middle-income countries.

**Revised** **as suggested**

- Introduction - Second Paragraph: The sentence "According to the Pakistan Demographic Health Survey (PDHS, 2018), almost 38% of children under five years of age are stunted, 23% are underweight, and 7% are wasted" needs a reference.

**Reference number 10 is provided at line number 10.**

  

- The words developing countries and LMICs should be used consistently. In the current form, both have been used interchangeably.

**Revised** **as suggested**

  

- The IYCF acronym was introduced in the first paragraph of the Introduction, and still used in full several times in the Introduction.

 
**Revised** **as suggested**

- Abstract - methods: The date of study mentioned was May to June 2018. However in the Introduction, it was mentioned that it was piloted in 2016 and evaluated between April to June 2018. Please check the dates and provide the correct one.

 
**Revised** **as suggested**

- Under mobile applications - Second Paragraph: Please add "study" in the sentence: "Consent to be a part of the was also obtained for participating in the project,"

 
**Added** **as suggested**

- Please provide the name of the smartphone application that was created?

**The name of application was “mhealth” and is mentioned in the text.**

  

- It would be more informative, if the interface of the application is attached to the paper and the type of options available for the participants to receive information on IYCF.

**Regarding the interface of the application, we have mentioned under heading of software availability that “the source code of android phone-based application developed for the Lady Health Workers (LHWs) under the project “Sehatmnd Kl” is the property of Maternal, Neonatal and Child Health Research Network (MNCHRN) and cannot be made public.”**

  

- Discussion: Please indicate if there is agreement of the results with the earlier studies?

**The text has been highlighted under the heading of discussion.**
